# Metal in biological samples from electronic cigarette users and those exposed to their second-hand aerosol: a narrative review

**DOI:** 10.3389/fmed.2024.1349475

**Published:** 2024-05-22

**Authors:** Diane Rezende Batista, Liana Sousa Coelho, Suzana Erico Tanni, Irma de Godoy

**Affiliations:** São Paulo State University (Unesp), Medical School, Botucatu, Department of Internal Medicine, Pulmonology Division, São Paulo, Brazil

**Keywords:** e-cigarettes, electronic cigarettes, vape, metal, heavy metals, hair, urine

## Abstract

**Introduction:**

Electronic nicotine delivery systems (ENDS) are gradually becoming more popular, particularly, among today’s youth. Despite being marketed as safe by the tobacco industry, the notable absence of regulation in their composition is evident. Both the generated fluids and aerosol exhibit a wide variety of substances that are not yet fully identified. In addition to additives, the aerosol contains metals, the presence of which can be attributed to the excessive heating of metallic filaments used in vaporizing the liquid.

**Objective:**

This review aimed to identify and describe studies that have assessed metal levels in biological samples obtained from electronic cigarette users and those exposed to their second-hand aerosol. This involved detailing the types and concentrations of metals identified and the biological samples in which the metals were detected.

**Methods:**

Two independent researchers conducted searches in the MEDLINE and EMBASE databases to identify studies that measured the metal levels in human non-invasive biological samples from electronic cigarette users and second-hand exposure. Data were presented as a narrative review.

**Results:**

In total, 18 articles were included in this review. Overall active and passive exposure to ENDS was related to higher levels of many metals, including lead and cadmium, in biological samples. ENDS users, in general, have lower metal concentrations in biological samples compared to the users of combustible cigarettes.

**Conclusion:**

The exposure to primary and second-hand e-cigarette aerosol is related to higher metal concentrations in the biological samples. The adverse effects of this exposure on long-term users are yet to be determined.

## Introduction

Electronic nicotine delivery systems (ENDS) were quickly accepted and have become very popular since their introduction in the United States in 2006. Recently, Cooper et al. reported a significant number of e-cigarette users among high school students (14.1%) and middle school students (3.3%) (1). In some European countries, the prevalence of e-cigarette use among teenagers has more than doubled in 4 years (2).

Although advertised as safe by the tobacco industry, there is no regulation on the form of use, concentration, and composition of aerosols in electronic devices. Furthermore, there are no guidelines on how manufacturers should report device characteristics and fluids available. The ban on the sales of menthol-flavored cigarettes became effective in Europe in 2020 (3) and in the USA in 2023 (4). However, there is no legislation for electronic cigarettes and hookahs. There are currently thousands of flavors for ENDS. In association with these flavors, fluid compositions for ENDS and the generated aerosols include a large, but not yet completely known, number of substances, whose effect and safety when used via inhalation are not defined.

In addition to additives, ENDS aerosol contains heavy metals. Metal aerosol in e-cigarettes are produced from vaporized fluid generated from the heating of metal filaments. These filaments are in general made from nichrome or kanthal (ferritic iron–chromium–aluminum alloy), so metals, such as silver (Ag), aluminum (Al), chromium (Cr), copper (Cu), iron (Fe), nickel (Ni), and zinc (Zn), are expected to be present in e-cigarette aerosols (5, 6). A systematic review published in 2020 (7) included 24 studies. In total, 12 studies detected metals/metalloids (Al, antimony, arsenic, cadmium, cobalt, Cr, Cu, Fe, lead, manganese, Ni, Se, tin, and Zn) in fluids and aerosols, and in 4 studies, metals were detected in samples of urine, saliva, serum, or blood from electronic cigarette users. An umbrella review (8) found evidence of these elements with considerable heterogeneity across the included studies. Two review studies examining EC users’ urine and serum showed similar or higher levels of metals and metalloids compared to samples of users of combustible cigarettes or cigars.

Information on metal concentrations in biological samples from e-cigarette users is scarce. A systematic review by Zhao et al. (7) included few studies where metal concentrations were available. In our systematic review, we have included 18 studies that measured metal/metalloid levels in human biological samples from electronic cigarette users and those exposed to their second-hand aerosol.

## Methods

**Figure 1:**
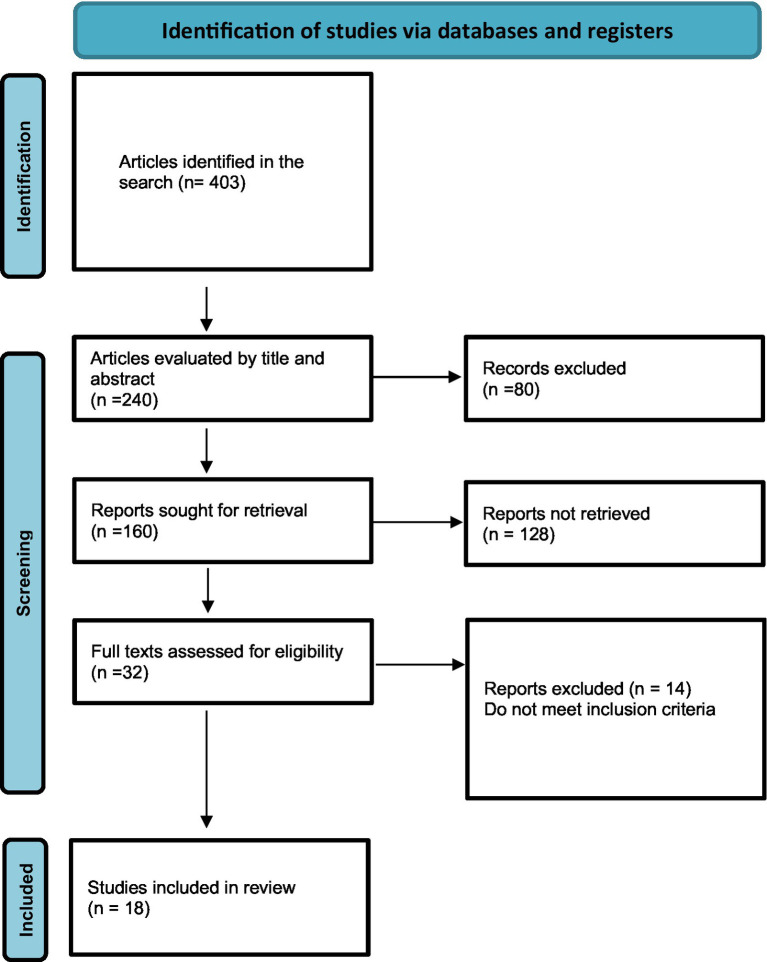
Preferred reporting items for systematic reviews and meta-analyses flow diagram of the process of including studies (9, 10).

For this narrative literature review regarding the identification and quantification of heavy metals in biological samples from electronic cigarette users, we performed a search of the MEDLINE and EMBASE databases in September and October 2023. The review was conducted according to Preferred Reporting Items for Systematic Reviews and Meta-analyses (PRISMA) recommendations (Figure 1).

The search strategy was formulated by two authors and approved by the rest of the group. The search strategies employed for PubMed were as follows: (Electronic Nicotine Delivery System OR Electronic Cigarettes OR E-Cigs OR E Cigs OR E-Cig OR E Cig OR E-Cigarettes OR E Cigarettes OR E-Cigarette OR E Cigarette OR Electronic Cigarette OR Cigarette, Electronic OR Cigarettes, Electronic OR THC Vaping OR THC Vapings OR Vaping, THC OR Vapings, THC OR E-Cig Use OR E Cig Use OR E-Cig Uses OR Use, E-Cig OR ECig Use OR ECig Uses OR Use, ECig OR Vape OR Vapes OR E-Cigarette Use OR E Cigarette Use OR E-Cigarette Uses OR Use, E-Cigarette OR Nicotine Vaping OR Nicotine Vapings OR Vaping, Nicotine OR Vapings, Nicotine OR Ecigarette Use OR Ecigarette Uses OR Use, Ecigarette OR Uses, Ecigarette OR Electronic Cigarette Use OR Cigarette Use, Electronic OR Electronic Cigarette Uses OR Use, Electronic Cigarette OR E Cigarette Vapor OR Vapor, E-Cigarette OR Electronic Cigarette Vapor OR Cigarette Vapor, Electronic OR Vapor, Electronic Cigarette) AND (Heavy Metals OR Heavy Metal OR Metal, Heavy OR Metal OR Metals) and for EMBASE (‘e cigarette’ OR ‘e cigarettes’ OR ‘electronic cigarettes’ OR ‘electronic nicotine delivery system’ OR ‘electronic nicotine delivery systems’ OR ‘electronic cigarette’) AND (‘metal, heavy’ OR ‘metals, heavy’ OR ‘heavy metal’).

Two researchers independently conducted article selection. Initially, the selection was based on article titles and abstracts. Subsequently, selected articles underwent full-text reading to determine their inclusion or exclusion in the review. Any discrepancies between the researchers were resolved through consensus or, when necessary, after discussion with a third researcher. The articles were selected according to established inclusion criteria, which involved the analysis of heavy metals in non-invasive biological samples from electronic cigarette users. The following studies were excluded: (1) review articles, (2) *in vitro* sample studies, (3) airway model studies, and (4) studies using animal samples. No restrictions were applied regarding publication date, language, or availability of full text during the selection process.

## Results

A total of 403 articles were identified in the selected databases. After removing duplicates, they were assessed based on the title and abstract. Review studies and those that did not meet the inclusion criteria, specifically those that did not address heavy metals and did not conduct biological sample analyses, were excluded. In total, 32 articles underwent comprehensive analysis of their full text. Following the exclusion of studies that did not meet inclusion criteria, 18 articles underwent detailed analysis and are described in this article.

Many studies have employed data from the Population Assessment of Tobacco and Health (PATH) Study, a longitudinal cohort study about tobacco use conducted among a sample of adults in the United States. Goniewicz et al. (11) analyzed data from users who only used e-cigarettes, who were dual users, and who never used any tobacco products from PATH Study Wave 1 (2013–2014). Urinary concentrations of Co, Pb, strontium, thallium, beryllium, Cd, and uranium were measured. A comparison between users who never used tobacco products and e-cigarette–only users showed Pb and Cd concentrations of approximately 19 and 23%, respectively, and were found to be lower in users who never used. The comparison between cigarette–only smokers and e-cigarette–only users showed Cd concentrations of 30% higher in in the first group. The comparison of the geometric mean of Pb and Cd concentrations between dual users and cigarette-only smokers did not differ.

In addition, from PATH Study Wave 1, Lizhnyak et al. (12) compared people who smoke against vape users and dual users split according to the frequency of cigarette and/or vape use. Urinary Cd levels were significantly different between people who frequently smoke and vape and people who frequently vape (0.33 vs. 0.28); between the group who infrequently smoke and vape (0.16) and people who smoke every day (0.31); and people who vape more than smoke (0.29) and people who frequently vape (0.28). There were no differences between groups for urinary Pb levels.

Dai et al. (13) evaluated changes in urinary heavy metal levels (Co, Mn, Be, Cd, Pb, Sr., Tl, and U) when users transitioned between cigarette, dual use, and no use. Switching from exclusive cigarettes or dual use to e-cigarettes or no use was not associated with a decrease in heavy metal levels in urine. Switching from exclusive e-cigarette use to exclusive cigarette use or dual use at follow-up was not associated with an increase in heavy metal levels in urine. In a similar study (14), people who transitioned from exclusive smoking to dual use, no significant changes in Pb concentrations were observed. Pb levels showed a significant decrease among dual users who transitioned to exclusive ENDS use, while other transition groups did not exhibit significant changes.

To evaluate whether exposure to certain biomarkers could be associated with some respiratory symptoms, Dai et al. categorized the participants into three different groups at baseline: non-users, e-cigarette-only users, and dual e-cigarette/tobacco users. Those reporting exclusive e-cigarette use or dual use at baseline presented a higher prevalence of respiratory illness symptoms in the past 12 months compared to non-users. In relation to urinary Cd and Cr levels, there were no differences between groups and no association with respiratory illness symptoms (15).

Kaplan, B et al. (16) analyzed urine samples collected during PATH Study Waves 1, 2, and 3 for Pb, Co, Mn, Cd, Be, Sr., Tl, and U. Out of the 173 current ENDS users in Waves 1, 2, and 3, 50 were exclusive ENDS users who had never used any other ENDS, and the users of Waves 1, 2, and 3 had a history of using non-electronic tobacco products, both combustible and non-combustible types, and had transitioned to becoming exclusive ENDS users. In exclusive ENDS users who never used any tobacco products, urinary Cd concentrations remained consistent across Waves 1, 2, and 3 (0.25, 0.20, and 0.35, respectively, *p* = 0.373). For users who never used any tobacco products, Cd concentrations were found to be 0.22, 0.22, and 0.23, respectively. In Wave 3, Cd levels were significantly higher in ENDS users who had not used other tobacco products compared to non-users of ENDS (*p* < 0.001) and all ENDS users across all waves (*p* < 0.001). Those who never used any tobacco products showed consistent Cd, Pb, Be, and Tl urinary concentrations across the three waves. After adjusting for various factors, such as demographics, passive smoke exposure, and substance use, the study found that the geometric mean ratios (GMRs) for urinary Cd and Pb concentration in exclusive ENDS users, former non-electronic tobacco users who switched to ENDS, and all ENDS users were higher than those of never users For other metals, GMRs were not significantly different for exclusive ENDS users who never used non-electronic tobacco products compared to non-users. The authors concluded that current exclusive ENDS users, who had never used any other non-electronic tobacco products between 2013 and 2016, exhibited higher levels of Cd and Pb in urine compared to those who never used any tobacco products.

Nathan et al. (17) analyzed data from adults of at least 21 years who provided their urine samples for the PATH Study Wave (5). Participants were categorized into four groups based on their past 30-day use of ENDS and cigarette smoking. The study showed that the geometric mean levels for all three metals (Cd, Pb, and U) were significantly higher among all tobacco users compared to non-users. Specifically, in those dual users who smoked <10 cigarettes/day, Cd levels were significantly lower compared to smokers. However, the levels in dual users who smoked ≥10 cigarettes/day when compared to exclusive smokers showed no significant difference.

Dai et al. (18) evaluated racial and ethnic disparities by analyzing PATH Study Waves 1–5 data and did not find differences in heavy metal (Cd and Pb) concentrations among non-Hispanic (NH) White people, NH Black people, Hispanic/Latino people, and NH other people.

In Spain, 100 participants (50 vapers, 25 dual users, and 25 non-tobacco smokers) were recruited, and samples of urine, hair, and exhaled breath condensate (EBC) were collected. In urine samples, only median Cr and Sn levels were significantly lower in controls than in vapers and dual users. In contrast, in hair, median Cr and Cd levels were significantly higher in controls than vapers and dual users. EBC samples presented metal concentrations below or close to the detection limit for the studied metals; therefore, an analysis was not possible (19).

Prokopowicz et al. (20) evaluated 90 volunteers who were stratified according to their use of tobacco. Analysis of urinary samples for Ba, vanadium (V), Ag, Mn, Co, Ni, Cr, Sb, Cd, and Pb found no significant differences in urine concentrations of these elements between e-cigarette users, non-smokers, and smokers. The same group of authors (21) also evaluated exposure to Cd and Pb in 156 volunteers who switched from cigarette to EC use. Blood Cd concentrations adjusted for age and gender were 0.31 (0.26–0.36), 0.44 (0.37–0.52), 1.38 (1.11–1.72), and 1.44 (1.16–1.78) μg/L in non-smokers, e-cigarette users, dual users, and smokers, respectively. *Post hoc* analysis revealed significantly lower Cd concentrations between non-smokers and users of any kind of tobacco product. Blood Pb concentrations were only significantly different between the non-smoker and smoker groups (*p* = 0.043).

Serum metal levels in a group of 150 Romanian individuals showed that Cu, molybdenum (Mo), and Zn levels were significantly higher in cigarette smokers. In addition, cigarette smokers had the highest concentrations of Sb and Sr. On the other hand, the highest concentrations of Ag, Se, and V were detected in e-cigarette users. Ni levels showed no differences between groups (22).

Using data from the 2013 and 2016 Korea National Health and Nutritional Survey, Lee et al. (23) evaluated a total of 4,744 participants (2,162 men and 2,582 women) who were categorized into five groups according to smoking and ENDS use habits. Cigarette smoking in men and women and E-cigarette use in men are associated with a higher risk of higher blood Cd levels. In men, urinary Cd levels were significantly higher in E-cigarette users than in non-smokers (past-smokers *p* = 0.017; cigarette-smokers *p* < 0.001).

A study that recruited 64 E-cigarette users (50 E-cigarette smokers who had never smoked or had quit smoking at least 3 months earlier) and 14 dual users (who used combustible cigarettes at least weekly) showed that compared to dual users, only e-cigarette users had higher urine Ni and Cr levels (24).

In a cross-sectional study (25), which evaluated urine samples for metal evaluation, it was found that Zn concentrations were significantly higher in electronic cigarette users than in non-smokers, but Zn concentrations in electronic cigarette users were not different when compared to cigarette smokers (470.7 ± 223.6 μg/g, *p* = 0.17).

Based on data extracted from NHANES 2015–2016, in the analyses of heavy metals adjusted for sex, race/ethnicity, age, and poverty levels, the relationship between current or former e-cigarette use and metals did not achieve statistical significance. Nevertheless, individuals with a history of smoking were found to be more prone to elevated levels of blood lead and urinary cadmium compared to those who had not used either e-cigarettes or traditional cigarettes (26).

Amalia et al. (27) conducted an observational study to evaluate environmental and individual exposure to second-hand e-cigarette aerosol (SHA) in two household types: e-cigarette user homes and control homes. In total, 77 participants were included: 29 exclusive e-cigarette users (exposed), 29 non-users, and 21 controls. They found 27 metals in urine samples. The concentrations of all urinary biomarkers were similar between non-users and control participants. Of metal concentrations analyzed in urine, Co showed a higher geometric mean concentration in non-users compared to control participants. Non-users living with e-cigarette users had significantly higher urine Co levels than non-users living in control homes. The authors concluded that e-cigarette use at home created bystander exposure to SHA, irrespective of the features related to the use of e-cigarettes. The same group of researchers (28) evaluated a family unit comprising an e-cigarette user, a pregnant woman who delivered an infant during the study, and their 3-year-old son. They found that metals were present in the urine and hair of all three participants, and also in the saliva of the adults, cord blood at the time of delivery, and breast milk. Several metals were identified in the urine, saliva, and hair samples of e-cigarette users, including Al, Cr, Ni, Cu, ZN, Sn, and Pb; however, Al was not found in urine. Metals were identified in cord blood and breast milk. Evaluation of samples from the 3-year-old revealed that the metals present in his urine and hair resembled those identified in samples from the pregnant woman, albeit generally in lower concentrations. Metals found at elevated concentrations in samples from the child, in contrast to those from the mother, included Zn in urine and Cr and Sn in hair. This research provided the first indications of involuntary exposure to e-cigarette aerosols in vulnerable populations, including children and pregnant women.

Table 1 lists heavy metals found in biological samples of electronic cigarette users.

**Table 1:** Metals identified in electronic smoking devices.

Human samples devices	Metal
Urinary concentrations	Pb, Cd, Be, Ni, Cr, and Co
Blood	Cd, Se, and V
Saliva	Cr, Ni, and Pb
Hair	Cr, Ni, and Pb

Pb, lead; Be, beryllium; Cd, cadmium; Co, cobalt; Cr, chromium; Se, selenium; V, vanadium; Ni-nickel.

## Discussion

This review shows that exposure to ENDS, active and passive, is associated with higher levels of several metals in biological samples. In addition, ENDS users, in general, present lower biosample metal concentrations compared to combustible cigarette users. Several metals have been evaluated in urine, blood, exhaled breath condensate, and hair samples. The adverse effects of the metals detected in biological samples from the reviewed studies are presented in Supplementary Table 1.

Nine (50%) of the studies included in this review are derived from the PATH Study, a longitudinal cohort study of tobacco use in a national sample of US adults, which evaluated metal concentrations in urine samples from different Waves. The following metal concentrations were evaluated: Be, Cd, Co, Pb, Sr., Tl, and U. Higher urinary Pb and Cd concentrations were found in e-cigarette smokers than in non-smokers by Goniewicz et al. (11) and Kaplan et al. (16). Nathan et al. (17) showed a significantly higher concentration of Cd, Pb, and U in smokers and ENDS users than non-users and dual exposure (cigarette smoke/ENDS) is associated with higher Cd levels and that there is an exposure–response relationship with the number of cigarettes per day. From the PATH Study Wave 1, Lizhnyak et al. (12) also found significantly higher urinary Cd levels in those who frequently smoke and vape than in those who only vape. Those who infrequently smoke and vape presented a significantly lower Cd concentration than people who smoke only cigarettes every day or frequently vaped and infrequently smoked. The urinary levels of Pb were similar in these groups. While PATH Study Waves were being conducted, there were changes in the generation of ENDS products; during the first waves, the earlier-generation products were predominant, while more recent-generation products were available during the most recent PATH Waves. However, the results are concordant over time.

Other small-scale studies in the USA and other countries produced results in line with the PATH-derived studies. Data from Romania showed significantly higher Cu, Mo, and Zn values in cigarette smokers than in non-smokers and EC users. Sb and Sr. concentrations were highest in cigarette smokers. In contrast, the highest concentrations of metals, such as Ag, Se, and V, were found in e-cigarette users (22). Sakamaki-Ching et al. (25), in the USA, showed that Se concentrations were significantly higher in electronic cigarette users than non-smokers and cigarette smokers, and Zn concentrations were significantly higher in electronic cigarette users than non-smokers. Lee et al. (23) analyzed data from the 2013 and 2016 Korea National Health and Nutritional Surveys and showed that regular cigarette smoking in men and women and ENDs use in men are associated with a higher risk of elevated blood Cd levels. In men, urinary Cd levels in electronic cigarette users were significantly higher than in non-smokers. A comparison between electronic cigarette smokers and dual users showed that exclusive e-cigarette users had higher urine levels of Ni and Cr (24).

Transition to different forms of smoking was also evaluated in participants of Waves 1 and 2 of the PATH Study (13). The transition from sole smoking to dual use showed no significant changes in Pb concentrations. However, there was a significant decrease in Pb level in dual users who transitioned to exclusive ENDS users. Prokopowicz et al. (21) also evaluated the exposure to Cd and Pb in 156 volunteers, and *ad hoc* analysis revealed significant differences in Cd concentration between non-smokers and electronic cigarette users and between non-smokers or electronic cigarette users and dual users or smokers. The only significant difference in blood Pb concentrations was observed between the non-smoker and smoker groups. The authors hypothesized that the exposure to Cd and probably to Pb could be significantly reduced by completely switching to ECs and quitting conventional cigarette smoking.

The influence of racial and ethnic disparities was also evaluated in PATH Study Waves 1–5 participants, with results showing no differences in Cd and Pb concentrations for non-Hispanic (NH) White people, NH Black people, Hispanic/Latino people, and NH other people (18).

One study that examined a sample subset from the PATH Study Waves 1 and 2 designed to evaluate exposure and respiratory symptoms found no differences between groups (non-users, exclusive e-cigarette users, and poly e-cigarette/tobacco users) in terms of urinary Cd and Cr levels, and also showed no association with respiratory symptoms (15). In addition, in contrast with most of the PATH-derived studies, data extracted from the NHANES 2015–2016 showed lower mean blood Pb and Cd levels in e-cigarette users (with or without dual use) when compared to sole e-cigarette users (current or former). Similar results were shown for Ba and Sb levels. Lower median levels in current or former e-cigarette users failed to reach statistical significance (26). Prokopowicz et al. (20) found no significant differences in urinary Ba, V, indium (In), Ag, Mn, Co, Ni, Cr, Sb, Cd, and Pb in people smoking different combinations of conventional and e-cigarettes.

Evaluation of environmental and individual exposure to second-hand e-cigarette aerosol (SHA) (27) showed that non-users living with ENDs users had significantly higher urine Co levels than non-users residing in control homes. Although the concentration is considered low, it is a marker of exposition.

The authors concluded that using e-cigarettes at home leads to exposure to SHA regardless of the characteristics of e-cigarette use. The same group of researchers, Ballbè et al. (28), evaluated homes with an e-cigarette user, a pregnant woman who delivered an infant during the study, and their 3-year-old son. Several metals were identified in the samples of e-cigarette users in urine, saliva, and hair, including Al, Cr, Ni, Cu, ZN, Sn, and Pb. However, Al was not found in urine. In the pregnant woman, several metals were found in cord blood and breast milk. Assessment of samples from the 3-year-old showed that metals found in his urine and hair were similar to those found in the pregnant woman, but usually at lower concentrations. Concentrations of Zn in urine and Cr and Sn in hair from the 3-year-old were higher than those in the mother. This study provided the first evidence of passive exposure to e-cigarette aerosols by people from vulnerable populations, such as children and pregnant women.

It is important to show that results may vary depending on the biological samples collected. A small study of urine, hair, and exhaled breath condensate samples showed that only median Cr levels in urine were significantly lower in controls than in vapers and dual users, whereas levels in hair were significantly higher in controls than in vapers and dual users. Exhaled breath condensate samples presented metal concentrations below or close to the detection limit for the metals studied, thus an analysis was not possible (19).

This review has some limitations. Half the data were derived from the PATH Study Waves, and data about the ENDS generation and other possible types of exposure to metals were not available in the different waves. In addition, most of the studies used self-reported data to classify categories of smokers. However, most of the results confirmed higher levels of metals in electronic cigarette users than non-smokers, with other smaller studies reinforcing this finding.

In conclusion, this review consistently shows that exposure to primary and second-hand e-cigarette aerosol is associated with higher concentrations of metals in biological samples in electronic cigarette users than non-smokers. It also shows that conventional combustible cigarette users have similar or higher metal levels than electronic cigarette users. Although the adverse effects of this exposure for long-term users are yet to be determined, further research related to the chemical characteristics of electronic cigarettes and their consequences in humans is urgently needed.

## Data availability statement

The original contributions presented in the study are included in the article/Supplementary material, further inquiries can be directed to the corresponding author.

## Author contributions

DB: Conceptualization, Data curation, Methodology, Writing – original draft, Writing – review & editing. LC: Data curation, Writing – original draft, Writing – review & editing. ST: Writing – review & editing. IG: Conceptualization, Data curation, Supervision, Writing – original draft, Writing – review & editing.
