# Supplementary material for: Metal in biological samples from electronic cigarette users and those exposed to their second-hand aerosol: a narrative review
Source: Front Med (Lausanne). 2024 May 22;11:1349475. doi: 10.3389/fmed.2024.1349475 (PMC11150601; doi:10.3389/fmed.2024.1349475)
Supplement: Supplementary file 1 [file Table_1.pdf]

Supplement 1

Table 1. Toxic effects and cancer probability of the principal metals found in biological samples from people who smoke or are exposed to electronic cigarettes (1)(2).

**Metal**	**Toxic effects** (ATSDR ToxGuide)	**Cancer probability** (IARC classification)
Cadmium	Renal tubular damage, glomerular impairment, bone demineralization, impaired lung function, emphysema	1
Lead	Reduced cognitive function, altered neuromotor and neurosensory function, reduced glomerular filtration rate, increased blood pressure, reduced sperm count, spontaneous abortion, reduced hemoglobin levels	2A
Beryllium	Acute beryllium disease (nasopharyngitis, dyspnea and chemical pneumonitis); Chronic beryllium disease (lung granulomas, respiratory symptoms, and impaired lung function)	1
Selenium	Nausea, vomiting, nail loss, hair loss, tachycardia	3
Chromium (VI)	Nasal and lung irritation and impaired pulmonary function	1
Vanadium	Damage to the lungs, throat and nose in rats, lung cancer	2B
Cobalt	Asthma-like allergy and decreased lung function, nose and throat irritation, erythrocytosis	2B
Nickel	Asthma, Lung inflammation, Contact dermatitis	2B
Uranium	Damage to kidney tubular and the respiratory tract following chronic inhalation	1
Molybdenum	Nasal lesions, decreases in renal function, liver damage	2B

ATSDR: U.S. Agency for Toxic Substances and Disease Registry. IARC (International Agency for Research on Cancer) classification: 1 - Carcinogenic to humans; 2A - Probably carcinogenic to humans; 2B - Possibly carcinogenic to humans; 3 - Not classifiable as to its carcinogenicity to humans

References:

1. ToxGuides™ - Letra A | ATSDR [Internet]. [cited 2023 Dec 1]. Available from: https://wwwn.cdc.gov/TSP/ToxGuides/ToxGuidesLanding.aspx
2. IARC classifications - National Cancer Control Policy [Internet]. [cited 2023 Dec 1]. Available from: https://wiki.cancer.org.au/policy/IARC_classifications
